# Emergency medicine residents’ learning curve in diagnosing deep vein thrombosis with 3-point venous point-of-care ultrasound

**DOI:** 10.1186/s12245-024-00645-x

**Published:** 2024-06-17

**Authors:** Soo Yeon Kang, Ik Joon Jo, Sejin Heo, Hansol Chang, Guntak Lee, Jong Eun Park, Taerim Kim, Se Uk Lee, Min Ji Kim, Hee Yoon

**Affiliations:** 1 Department of Emergency Medicine, Chung-Ang University Gwangmyeong Hospital, Chung- Ang University School of Medicine, Gwangmyeong, Gyeonggi-do 14353 Republic of Korea; 2 Department of Emergency Medicine, College of Medicine, Kangwon National University, Chuncheon, Gangwon-do 24341 Republic of Korea; 3 Department of Emergency Medicine, Samsung Medical Center, Sungkyunkwan University School of Medicine, 115 Irwon-ro, Gangnam-gu, Seoul, 06351 Republic of Korea; 4 Biomedical Statistics Center, Research Institute for Future Medicine, Samsung Medical Center, Seoul, 06351 Republic of Korea

**Keywords:** Emergency medicine, Deep vein thrombosis, Point-of-care ultrasound, Learning curve, Diagnostic accuracy

## Abstract

**Background:**

Many cases of deep vein thrombosis (DVT) are diagnosed in the emergency department, and abbreviated lower extremity venous point-of-care ultrasound (POCUS) has already shown an accuracy comparable to that of specialists. This study aimed to identify the learning curve necessary for emergency medicine (EM) residents to achieve expertise-level accuracy in diagnosing DVT through a 3-point lower extremity venous POCUS.

**Methods:**

This prospective study was conducted at an emergency department between May 2021 and October 2022. Four EM residents underwent a one-hour POCUS training session and performed DVT assessments in participants with DVT symptoms or confirmed pulmonary embolism. POCUS was performed at three proximal lower extremity sites to evaluate the thrombi presence and vein compressibility, with results validated by specialized radiology ultrasound. Cumulative sum (CUSUM) and the Bush and Mosteller models were used to analyze the learning curve, while generalized estimating equations were used to identify factors affecting diagnostic accuracy.

**Results:**

91 POCUS scans were conducted in 49 patients, resulting in 22% DVT confirmed by specialized venous ultrasound. In the CUSUM analysis, all four EM residents attained a 90% success rate at the common femoral vein, whereas only half achieved this rate when all three sites were considered. According to Bush and Mosteller models, 13–18 cases are required to attain 90–95% diagnostic accuracy. After 10–16 cases, the examination time for each resident decreased, and a 20% increase in examiner confidence was linked to a 2.506-fold increase in the DVT diagnosis accuracy.

**Conclusion:**

EM residents generally required 13–18 cases for 90–95% DVT diagnostic accuracy, but proficiency varied among individuals, particularly requiring more cases for regions outside the common femoral vein.

## Introduction

Venous thromboembolism (VTE) is the third most common cardiovascular disease and primarily manifests in two forms: deep vein thrombosis (DVT) and pulmonary embolism (PE) [1, 2]. A 2018 study showed a rise in South Korea’s VTE incidence, with DVT and PE cases per 100,000 increasing from 8.1 to 13.2 in 2009 to 12.7 and 16.6 in 2013, reflecting an ongoing annual increase [3]. Importantly, untreated DVT can progress to PE in 30–60% of cases, thereby elevating mortality rates [4, 5]. Given that the emergency department (ED) diagnoses more than half of all VTE cases [6, 7], timely and accurate diagnosis by emergency medical personnel is crucial.

DVT commonly originates in the leg veins. Traditional clinical signs such as Homan’s sign, edema, and tenderness are not reliable indicators of DVT because of their non-specific nature [8, 9]. Venography, once considered the gold standard for diagnosis, is invasive and can be painful [10, 11]. Computed tomography carries risks such as radiation exposure and the potential for insufficient contrast enhancement [12]. Currently, non-invasive lower extremity venous ultrasound has become the primary diagnostic method for DVT. Traditional whole-leg venous ultrasound is an exhaustive evaluation of the femoral and popliteal veins and their branches, demonstrating a sensitivity of 91–96% and a specificity of 98–100% [13–15]. However, referring patients to specialized radiology ultrasound laboratories for whole-leg ultrasound can result in delays, as many of these laboratories do not operate 24/7, compelling patients to remain in the ED until standard operating hours.

Recently, the application of point-of-care ultrasound (POCUS) performed by emergency physicians has expanded [16, 17], and various studies have focused on its efficacy in diagnosing lower extremity DVT. Notably, abbreviated lower extremity venous POCUS, conducted at the bedside by emergency physicians at two or three specific sites, has demonstrated an accuracy comparable to that of specialists [12, 18–21] and is significantly faster [21–23]. Despite these advantages, there is a research gap in defining the ultrasound experience level required by emergency physicians to achieve specialist-level accuracy. This study aimed to address this gap by identifying the learning curve necessary for emergency medicine (EM) residents to achieve expertise-level accuracy in diagnosing DVT using a 3-point lower extremity venous POCUS.

## Materials and methods

### Study overview

This prospective observational study was conducted in the ED of an urban academic hospital with an annual volume of 70,000 people in South Korea from May 2021 to October 2022. All participants provided informed written consent, and the study was approved by the Institutional Review Board (IRB) of the Samsung Medical Center (IRB number: 2021-01-128-002).

### Study population

#### Patients

The study included adult patients aged 18 years and older who presented with symptoms of lower extremity suggestive of DVT or had confirmed PE requiring DVT assessment. We excluded patients diagnosed with DVT before ED arrival, hemodynamically unstable not suitable for transport to specialized radiology ultrasound laboratories, with central venous catheters in the femoral vein that precluded examination, and who declined participation.

### Ultrasound examiners

Four second-year EM residents working in tertiary academic ED participated in this study. Each resident underwent abdominal and cardiac ultrasound training, with experience in performing approximately 100–150 POCUS scans. However, they had no prior training or experience in lower extremity venous ultrasound.

### Study protocol

The participating EM residents underwent a one-hour training on lower extremity venous ultrasound, combining a PowerPoint lecture and practical scanning. In the ED, the resident conducted a 3-point lower extremity venous ultrasound examination. Subsequently, the patient was referred to our institution’s radiology ultrasound laboratory for specialized venous ultrasound, which served as the standard for accurate examination. The study utilized a 7–16 MHz linear probe of the Samsung Ultrasound HM70A model (Samsung Medison, Seoul, South Korea) at the ED.

**Fig. 1:**
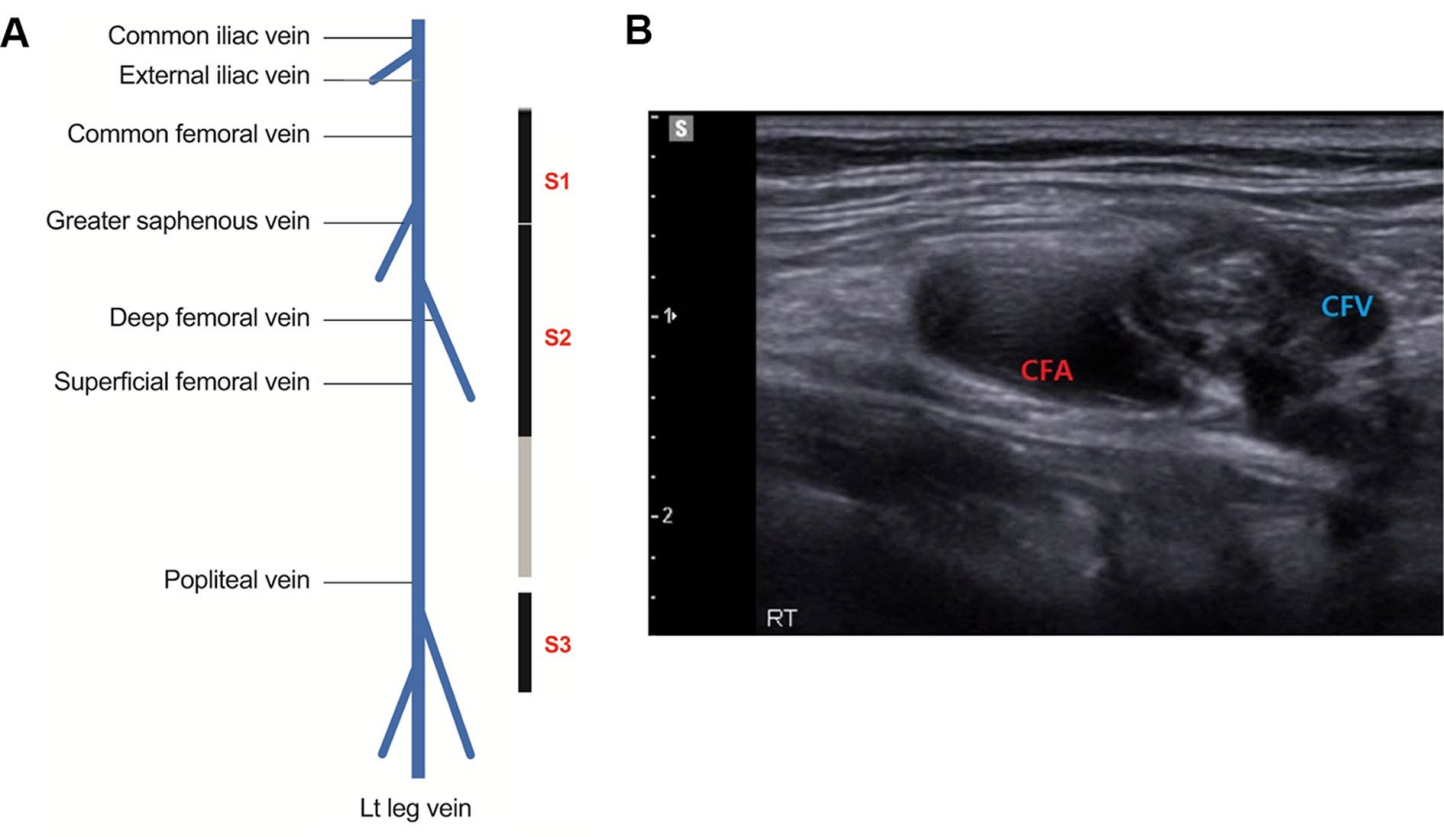
Protocol for 3-point Lower Extremity Venous POCUS Scan. **A**. The 3-point POCUS approach targets the following vascular landmarks: from superior to a common femoral vein to the great saphenous vein bifurcation (S1), proximal superficial and deep femoral vein segment (S2), and popliteal vein to the trifurcation (S3). Black bars denote mandatory scanning areas for POCUS. A gray bar indicates an additional region that requires more scanning effort. **B**. An instance of a non-compressible common femoral vein due to thrombosis, identified during probe compression. *Abbreviations POCUS* point-of-care ultrasound; *CFA* common femoral vein; *CFV* common femoral vein

This study conducted POCUS examinations at three proximal sites on the lower extremities. DVT was determined based on two criteria assessed via ultrasound: (1) direct observation of the presence or absence of thrombi, (2) complete compressibility of the vein when pressure was applied with the ultrasound probe [12, 20]. The first site (Site 1, S1) was the section from the common femoral vein to the confluence of the great saphenous vein. During scanning, pressure was repeatedly applied and released while sliding slowly along the vein to observe whether the lumen had completely disappeared. The second site (Site 2, S2) extended below S1 toward the direction of the knee, continuing the scan until both the superficial and deep femoral veins branched off and were no longer visible on ultrasound. The third site (Site 3, S3) involved scanning approximately 2–3 cm of the popliteal fossa area from the popliteal vein to the trifurcation (Fig. 1A). During S3 scanning, accessibility to the popliteal vein was improved by flexing the knee and externally rotating the hip. A positive finding of DVT was defined as a visualized thrombus or incomplete compression of the vein, and a negative was defined as no thrombus and complete collapse of the vein lumen upon probe compression (Fig. 1B). EM residents were instructed to make one of three judgments: “negative,” “positive,” or “inconclusive.”

### Data collection

Data were collected on the following variables for each participant: age, sex, body mass index (BMI), medical history, lower limb symptoms, and D-dimer values. The Wells scores were calculated using these data. Additionally, EM residents documented the time taken for the POCUS scans. After the lower extremity venous POCUS scan, a final judgment was made by noting the presence or absence of thrombi and compressibility. The confidence level of the EM residents’ assessment after each POCUS was recorded on a six-point scale from 0 to 100% at 20% intervals.

### Outcome measures

The primary outcome was the number of lower extremity venous POCUS examinations required by EM residents to achieve a diagnostic accuracy of 90% or more compared with the final diagnosis by specialized venous ultrasound. Secondary outcomes included the learning curve for each point on the POCUS examination, factors affecting the accuracy of the ultrasound examination, and the derivation of a learning curve based on the reduction in the duration of the POCUS examination.

### Statistical methods

#### Descriptive statistics

Standard descriptive statistics were used for the quantitative analysis of the collected statistical data. The Chi-square test or Fisher’s exact test was used to compare categorical variables, whereas one-way ANOVA or the Kruskal–Wallis test was employed to compare continuous variables at the patient level among the four EM resident groups. The variables collected at the exam level at the three sites were compared using a generalized regression model with the generalized estimating equations (GEE) approach accounting for the correlated data.

#### Sample size calculation and the learning curve

The learning curve of lower extremity venous ultrasound examinations for accurate DVT diagnosis is determined by calculating the cumulative sum (CUSUM) statistics, using parameters such as acceptable failure rate (*p*_0_) = 10%, unacceptable failure rate (*p*_1_) = 30%, type 1 error (α) = 0.05, and type 2 error (β) = 0.2 [24]. We defined success as agreement of the final diagnosis between POCUS and specialized venous ultrasound, and failure as disagreement. The minimum sample sizes for the acceptable and unacceptable failure rates were 14 and 17, respectively. After adjusting for 20% addition to the minimum sample size for the unacceptable failure rate and accounting for a 10% dropout rate, we determined a total of 22 cases per examiner. The CUSUM statistics in learning curves are categorized based on the CUSUM line’s final position: above the upper control limit (insufficient learning), between the control limits (undefined performance), or below the lower control limit (sufficient learning). The number of cases required to achieve success rates (diagnostic accuracy) of 90% and 95% were estimated using the predicted learning curve based on the Bush and Mosteller learning model, defining the expected probability of success as 0.9 and the expected probability of failure as 0.3 [24, 25]. The learning curve for the ultrasound scan time was plotted as a CUSUM graph by calculating the sequential difference between the raw data and the mean value.

#### Factors affecting success rate

Multivariable analysis aimed to identify factors significantly influencing the overall success rate. We utilized a generalized regression model using the GEE approach to account for correlated data. Factors affecting the success rate included individual EM residents, patient BMI, D-dimer values, Wells score, and the examiner’s confidence in their ultrasound examination.

Statistical analysis was performed using SAS version 9.4 (SAS Institute, Cary, NC, USA), and a p-value less than 0.05 was considered statistically significant.

## Results

### Characteristics of the study population

In the study, 91 lower extremity venous POCUS examinations were performed on 49 patients, with each EM resident conducting 22–23 examinations. Of the patients, 47% were male (*n* = 23) with a median age of 73, and common conditions included malignancy, hypertension, and diabetes. Edema was noted in 61.5% of cases (*n* = 56). Specialized ultrasound confirmed lower extremity DVT in 22% of cases (*n* = 20), and other conditions like intra-abdominal tumors, leg hematomas, abscesses, and peripheral arterial stenosis were also differentiated (Table 1).

**Table 1:** Baseline characteristics of the study population

Patients (*N* = 49)		Exams (*N* = 91)	
Male, N (%)	23 (46.9)	Leg symptoms, N(%)	
Age, median (IQR)	73 (61, 80.5)	Asymptomatic	16 (17.6)
BMI, mean ± SD	24.1 ± 3.61	Edema	56 (61.5)
Past medical history, N (%)		Pain	16 (17.6)
HTN	23 (46.9)	Warmth	4 (4.4)
DM	18 (36.7)	Redness	6 (6.6)
Coronary disease	3 (6.1)	Tenderness	6 (6.6)
Chronic lung disease	2 (4.1)	Final diagnosis of exams, N(%)	
CVD	2 (4.1)	No DVT in 3 sites	71 (78)
Malignancy	25 (51)	-No abnormal findings	48
Past DVT history	2 (4.1)	-DVT in distal sites	14
Past PE history	0 (0)	-Artery problem	3
Current PE	6 (12.2)	-Other causes	6
Immobilization	5 (10.2)	DVT in 3 sites	20 (22)
Pregnancy	1 (2)	-Only 3 sites	7
D-dimer, median (IQR)	5.62 (1.72, 14.27)	-combined other sites	13

Data are presented as n (%) or mean ± SD, median (IQR). *Abbreviations IQR* interquartile range; *BMI* body mass index; *SD* standard deviation; *HTN* hypertension; *DM* diabetes mellitus; *CVD* cerebrovascular disease; *DVT* deep venous thrombosis; *PE* pulmonary embolism

### Comparative assessment among EM residents

In the patient cohort evaluated by four EM residents, no significant differences were found in sex, age, or BMI. However, the time taken for ultrasound scans varied notably among the residents (*P* < 0.001). Regarding accuracy, the residents made 2, 1, 4, and 4 misjudgments each. There was also a significant difference in the confidence levels each resident reported in their assessments (*P* < 0.001) (Table 2).

**Table 2:** Comparison of demographic characteristics and final diagnoses among four emergency medicine resident groups

Patient Level	EM 1 (*n* = 13)	EM 2 (*n* = 12)	EM 3 (*n* = 13)	EM 4 (*n* = 11)	*P*-value
Male sex, N (%)	7 (53.8)	4 (33.3)	6 (46.2)	6 (54.5)	0.706
Age, median (IQR)	78 (73, 83)	68 (65, 75.5)	66 (59, 76)	75 (66, 81)	0.236
BMI, mean ± SD	23.8 ± 2.31	24.6 ± 4.62	24.7 ± 3.50	23.0 ± 3.88	0.640
Immobilization within 3 months, N (%)	0	0	4 (30.8)	1 (9.1)	0.026
US scan time (minutes)	2.96 ± 1.3	5 (5, 7)	3 (2, 6)	4 (3, 4)	< 0.001^*^
**Exam Level**	**EM 1** **(n = 23)**	**EM 2** **(n = 23)**	**EM 3** **(n = 23)**	**EM 4** **(n = 22)**	**P-value**
Final Diagnosis -no DVT in 3 sites -DVT in 3 sites	21 (91.3) 2 (8.7)	18 (78.3) 5 (21.7)	16 (69.6) 7 (30.4)	16 (72.7) 6 (27.3)	0.605
Overall success	21 (91.3)	22 (95.7)	19 (82.6)	18 (81.8)	0.419
Success in site 1	21 (91.3)	23 (100)	20 (87.0)	20 (90.9)	0.848
Success in site 2	22 (95.7)	19 (82.6)	19 (82.6)	19 (86.8)	0.549
Success in site 3	20 (87.0)	21 (91.3)	20 (87.0)	18 (81.8)	0.854
Confidence 0–20 40–60 80–100	- 9 (39.2) 14 (60.9)	1 (4.4) - 22 (95.7)	2 (8.7) 2 (8.7) 19 (82.6)	1 (4.6) 2 (9.2) 19 (86.3)	< 0.001^*^

Sex was assessed using the Chi-square test. Age was evaluated using the Kruskal–Wallis test. BMI was analyzed using one-way analysis of variance (ANOVA) and immobilization data were assessed using Fisher’s exact test. Other data were processed using a generalized estimating equation. *P value denotes significance. *Abbreviations EM* emergency medicine; *IQR* interquartile range; *BMI* body mass index; *SD* standard deviation; *US* ultrasound; *DVT* deep venous thrombosis

### Deriving learning curves for lower extremity POCUS

#### Overall success rate

**Fig. 2:**
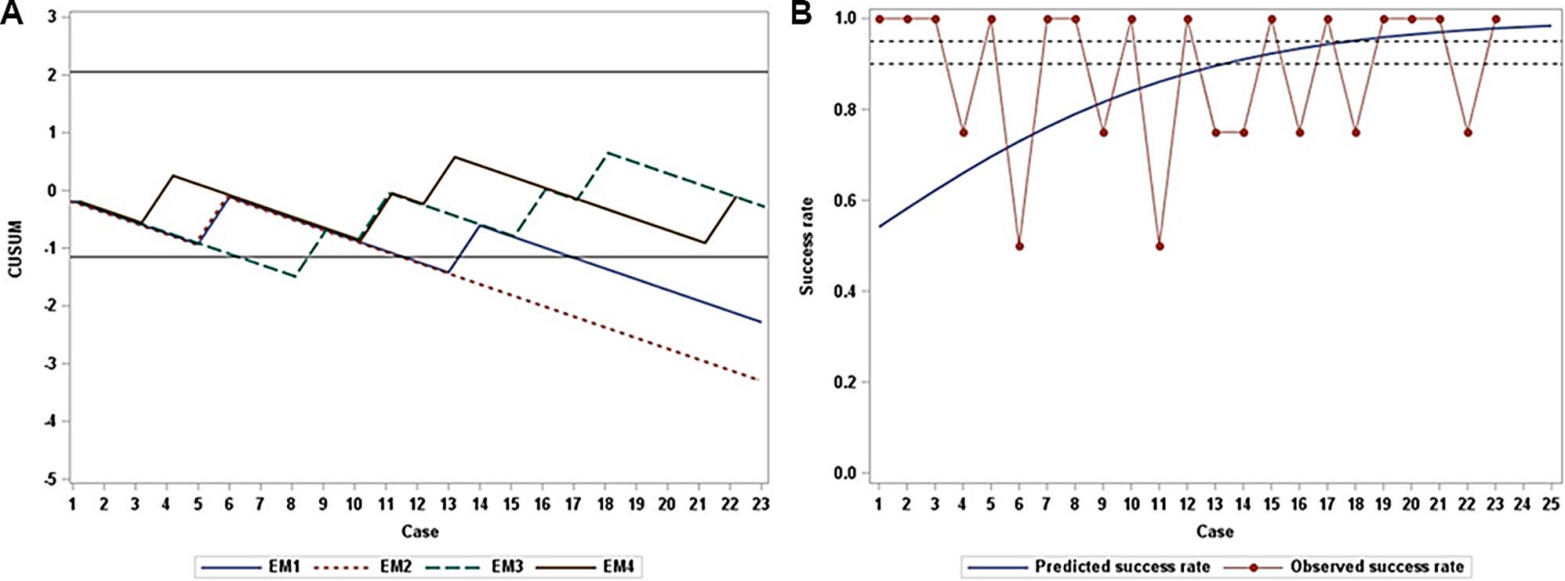
Learning Curves Using CUSUM Graphs and the Bush and Mosteller Learning Model. **A**. Learning curves depicted using CUSUM. Each line represents the learning curve of each EM resident. An increasing trend indicated failure, whereas a decreasing trend indicated success. Horizontal black lines indicate control limits: 2.05 as the upper control limit and − 1.15 as the lower control limit. **B**. The observed success rate (depicted by the red connected line) versus the predicted success rate (blue line) for each case based on the Bush and Mosteller learning model. The predicted 90% success rate was achieved in approximately 13 cases. The dotted lines represent 90% and 95% success rates, respectively. *Abbreviations CUSUM* cumulative sum; *EM* emergency medicine

As illustrated in Fig. 2A, EM residents 1 and 2 surpassed the lower control limit (-1.15), thereby demonstrating significant proficiency in their assessments. Conversely, EM resident 3 crossed the lower control limit in the sixth case, but remained within the control limits in subsequent cases, making the evidence for proficiency statistically insignificant. EM resident 4 exhibited similar results. Importantly, none of the residents exceeded the upper control limit (2.05), suggesting that none of them were unskilled. However, only EM residents 1 and 2 achieved success rates exceeding 90%, when all three sites were considered. According to the Bush and Mosteller learning model, the number of cases required to reach a 90% success rate was 13, whereas achieving a 95% success rate required 18 cases (Fig. 2B).

#### Success rate by examination site

**Fig. 3:**
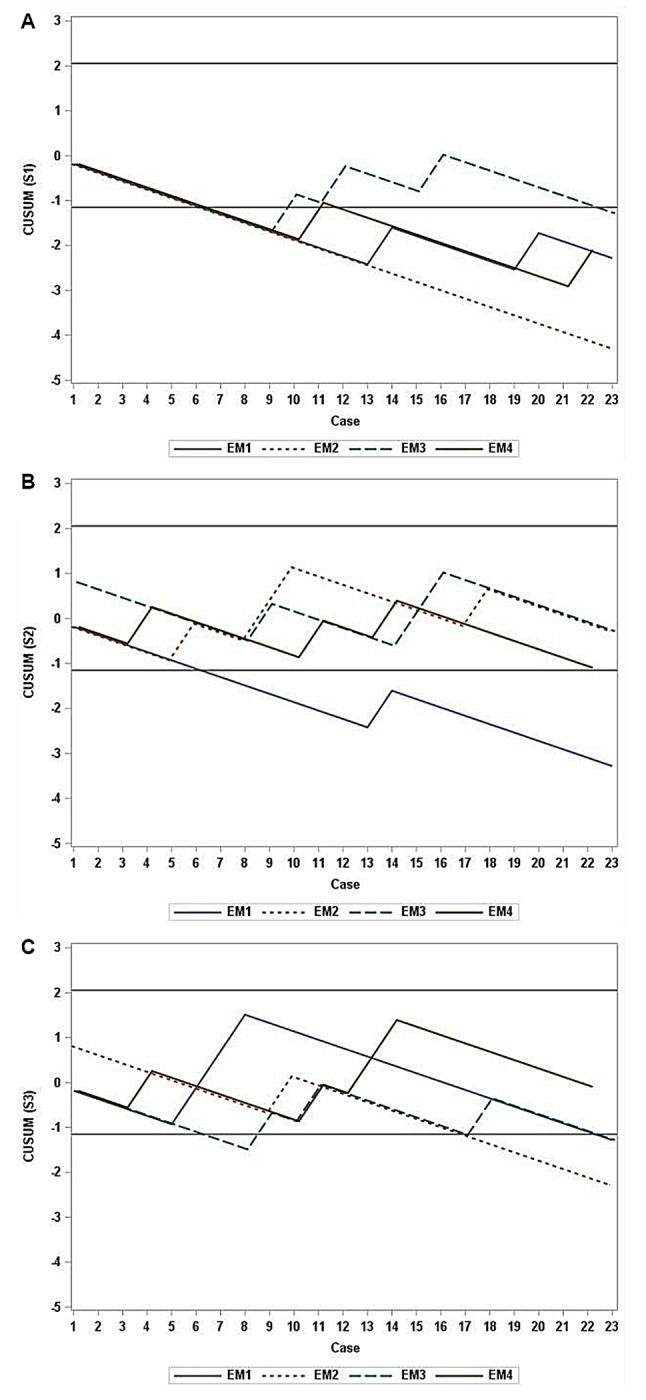
Learning Curves Using CUSUM Graphs by Exam Site. **A**. Learning curves shown using CUSUM graphs at the femoro-saphenous junction (S1). **B**. Learning curves displayed using CUSUM graphs at the femoral vein (S2). **C**. Learning curves portrayed using CUSUM graphs at the popliteal fossa (S3). *Abbreviations CUSUM* cumulative sum

The learning curves for each of the three locations using lower extremity POCUS are presented in Fig. 3. All four residents achieved a 90% success rate at S1 (Fig. 3A); only EM resident 1 achieved success at S2 (Fig. 3B), and EM resident 4 did not achieve success at S3 (Fig. 3C).

### Proficiency assessment through scan time reduction using CUSUM

**Fig. 4:**
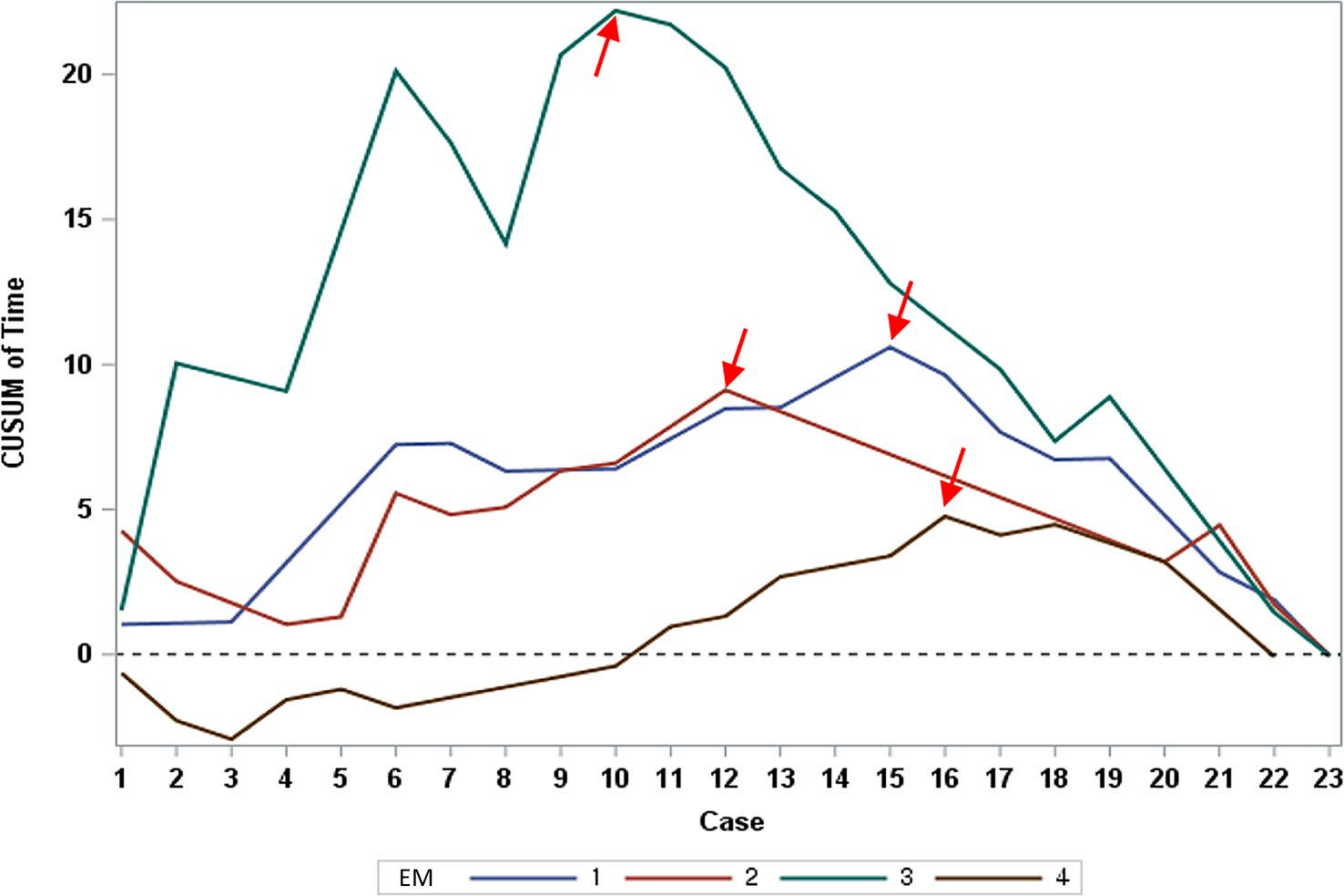
CUSUM Plot for Time. Learning curves using the CUSUM method for scan time versus case number. Each line represents each emergency medicine resident’s learning curve for the scan time. *Abbreviations CUSUM* cumulative sum

Arrows in Fig. 4 mark points where ultrasound scan times decreased for each EM resident, indicating improved proficiency. Reductions occurred after the 15th, 12th, 10th, and 16th examinations for each resident. These findings suggest that between 10 and 16 examinations are needed to achieve decreased scan times due to increased proficiency.

### Analysis of factors affecting success rate

A multivariable analysis was conducted to evaluate the impact of factors like EM resident identity, patient BMI, D-dimer levels, Wells scores, and assessment confidence level on the success rate of lower extremity venous POCUS. The analysis revealed that all factors except confidence level had no significant effect on the accuracy of diagnoses. Notably, with every 20% increase in a resident’s confidence level, the odds of an accurate DVT diagnosis rose by 2.506 times (OR = 2.506, 95% CI = 1.317–4.767) (Table 3).

**Table 3:** Multivariable analysis of risk factors for overall success

Variable	*p*-value	OR	95% CI of OR
EM (ref. 1)	0.147		
EM 2	1.000†	0.684	0.033–14.001
EM 3	0.622†	0.151	0.004–5.443
EM 4	0.307†	0.144	0.008–2.461
BMI	0.990	0.998	0.742–1.342
D-dimer	0.389	0.978	0.930–1.029
Wells score ≥ 3 (ref. <3)	0.827	1.285	0.135–12.262
Confidence	0.005^*^	2.506	1.317–4.767

All the data were analyzed using a generalized estimating equation. The †P value was adjusted using the Bonferroni correction for post-hoc comparisons. A *P value denotes significance. *Abbreviations EM* emergency medicine; *ref* reference; *BMI* body mass index; *OR* odds ratio; *CI* confidence interval

## Discussion

Accurate lower extremity DVT diagnosis and treatment are critical due to the risk of potentially fatal PE. While tools like the Wells score and D-dimer tests aid in assessing DVT risk [26, 27], imaging, particularly ultrasound, is crucial for confirming suspicions [28]. Despite being the preferred diagnostic method, its implementation is often delayed due to the requirement for trained specialists. Thus, the American College of Emergency Physicians (ACEP) emphasizes the importance of POCUS examinations performed directly by emergency physicians in high-risk patients and the need for ultrasound education [29, 30]. A notable research gap exists in determining the experience level emergency physicians need for specialist-level accuracy. Our study addresses this by quantifying the training EM residents require to diagnose DVT using ultrasound proficiently. We found that 13 cases were needed to achieve a diagnostic accuracy of 90%, and 18 cases had 95% accuracy in lower extremity venous POCUS examinations. These results provide foundational data for emergency physicians to effectively utilize lower extremity ultrasound in clinical settings.

The 2008 ACEP guidelines recommend residents complete at least 20 h of didactic education and 150 hands-on scans, including 25–50 scans per application [17, 30]. Our study, however, provided just one hour of lower extremity venous POCUS training, comprising a 40-minute lecture and 20-minute hands-on session, with no pre-study patient practice. This brief training, shorter than the 2–5 h in prior studies [18–21, 31, 32], aimed to accurately gauge the learning curve without prior ultrasound experience influencing it. Despite this, our study demonstrated that participants reached a 90% success rate after completing just 13 scans, indicating that minimal training might be sufficient for effectively implementing lower extremity venous POCUS in emergency care. This efficiency is notable compared to the recommended 20 and 25 cases for appendicitis and cholecystitis learning [33, 34].

In this study, EM residents required 1 to 1.5 years to complete the necessary lower extremity POCUS examinations, each making a few incorrect judgments. The main causes of these errors were chronic partial thrombosis (*n* = 3), severe leg edema (*n* = 3), pain-related examination limitations (*n* = 2), and knee flexion restrictions (*n* = 2). This mirrors previous studies that reported errors due to factors like unclear ultrasound images in obese patients, unusual venous courses, chronic thrombi, and misidentifications of conditions such as lymph nodes or superficial thrombophlebitis as DVT [20–22, 32]. Crucially, seven errors occurred following extended intervals of 2–5 months between ultrasound exams, suggesting that infrequent practice increases failure rates. This highlights the critical need for regular practice to sustain ultrasound proficiency [20, 35].

Ultrasound is influenced by factors such as the patient’s BMI and the skill level of the ultrasound operator [36, 37]. In obese patients, effective ultrasound penetration can be challenging, making diagnosis difficult. According to a study by Dua A et al. [38]. , it was recommended to opt for alternative imaging studies when diagnosing DVT in patients with a BMI greater than 40. Another study indicated that incorrect judgments were significantly associated with higher BMI levels (34.7 vs. 28.5 kg/m^2^, *P* < 0.001) [20]. However, our study did not find a significant BMI impact on DVT diagnosis, possibly because our average patient BMI (24.1 ± 3.61) was not high enough to affect results.

Medical institutions differ in their specialized lower extremity venous ultrasound protocols, with some focusing on specific regions and others scanning the entire leg to avoid missing isolated distal DVTs. Despite debates regarding the clinical importance of treating isolated distal DVT [39, 40], anticoagulation is recommended in high-risk cases because distal DVT can progress proximally in up to 25% of cases [41, 42]. However, ultrasound sensitivity for asymptomatic distal DVT can decrease from 74 to 50% [43], and the examinations are time-intensive. Simplified protocols covering just the common femoral and popliteal veins might miss 8% of distal DVTs but offer similar long-term outcomes [44], making them practical choices for DVT POCUS. Our study’s learning curve analysis revealed that the common femoral region (S1) was the easiest to scan, while the extensive S2 region posed challenges in acquiring high-quality vessel images. According to Caronia J.’s study, the S1 region demonstrated 100% sensitivity and 97% specificity, but the sensitivity of the S3 region decreased to 78%, likely due to the smaller size of the vein and challenges in positioning due to hip trauma, obesity, edema, and contractures [32].

The examination time, recorded from when the probe touched the patient’s skin until the scan’s end, was a median of 4 min (IQR: 3–5 min), consistent with previous studies reporting around 3 to 5 min [20, 23]. In the fast-paced ED setting, completing ultrasound examinations in under 5 min is practical for EM doctors. A reduction in examination time, while retaining accuracy, indicates growing proficiency. As shown in Fig. 4, the examination time typically decreased after 10–16 scans, aligning with the 13–18 cases needed to reach 90–95% accuracy.

The EM residents’ confidence in their diagnoses reflects the varying difficulty of cases. When doctors were 100% confident, only one error occurred in the final judgment. In cases with a confidence level of 60% or lower, all residents except EM resident 1 made errors. Table 3 indicates that each 20% increase in confidence significantly raised the odds of a correct DVT diagnosis by 2.506 times. This implies that as EM residents become more confident through experience and training, their diagnostic accuracy improves.

Our study had several limitations. First, it was conducted at a single medical institution and involved a small number of second-year EM residents with varying levels of POCUS experience. In addition, the study was conducted at a tertiary emergency center with a high proportion of malignant and severely ill patients, limiting the generalization of our results. Therefore, it is necessary to conduct research in more diverse settings. Second, to minimize the impact of prior education on learning outcomes, we did not provide comprehensive pre-training in lower extremity venous POCUS, as recommended by the ACEP. The brief one-hour preliminary training session may not have been sufficient for effective learning. Therefore, this study did not evaluate the effectiveness of POCUS education per se; however, adequate preliminary education is expected to have resulted in fewer learning cases. Lastly, it took between 1 and 1.5 years for the participants to achieve the target examination count, with breaks ranging from 2 to 5 months. These inconsistent ultrasound examinations could have negatively influenced proficiency. In addition, the accumulation of ultrasound scanning techniques and clinical experience over the years may have influenced the judgments.

## Conclusion

This study explored the learning curve of EM residents performing three-point lower extremity venous POCUS examinations. EM residents generally required 13–18 cases for 90–95% DVT diagnostic accuracy, but proficiency varied among individuals, particularly requiring more cases for regions outside the common femoral vein. Continuous education and training are crucial to maintain proficiency in ultrasound skills.

## Data Availability

No datasets were generated or analysed during the current study.
